# Interaction of hydrophobic polymers with model lipid bilayers

**DOI:** 10.1038/s41598-017-06668-0

**Published:** 2017-07-25

**Authors:** D. Bochicchio, E. Panizon, L. Monticelli, G. Rossi

**Affiliations:** 1 Physics Department, University of Genoa, Via Dodecaneso 33, 16146 Genoa, Italy; 2 University of Lyon, CNRS, Molecular Microbiology and Structural Biochemistry, UMR 5086, 7 Passage du Vercors, F-69367 Lyon, France

## Abstract

The interaction of nanoscale synthetic materials with cell membranes is one of the key steps determining nanomaterials’ toxicity. Here we use molecular simulations, with atomistic and coarse-grained resolution, to investigate the interaction of three hydrophobic polymers with model lipid membranes. Polymer nanoparticles made of polyethylene (PE), polypropylene (PP) and polystyrene with size up to 7 nm enter easily POPC lipid membranes, localizing to the membrane hydrophobic core. For all three materials, solid polymeric nanoparticles become essentially liquid within the membrane at room temperature. Still, their behavior in the membrane core is not the same: PP and PS disperse in the core of the bilayer, while PE shows a tendency to aggregate. We also examined the interaction of the polymers with heterogeneous membranes, consisting of a ternary lipid mixture exhibiting liquid-ordered/liquid-disordered phase separation. The behavior of the three polymers is markedly different: PP disfavors lipid phase separation, PS stabilizes it, and PE modifies the topology of the phase boundaries and causes cholesterol depletion from the liquid ordered phase. Our results show that different hydrophobic polymers have major effects on the properties of lipid membranes, calling for further investigations on model systems and cell membranes.

## Introduction

As plastic production approaches 300 million tons per year, the most recent estimates suggest that from 5 to 13 million tons of plastic waste enter the ocean every year^1^. At moderate temperatures, solar radiation and oxidizing conditions^2^ progressively reduce the size of floating plastic fragments down to the micro and nano scale. During the last decade researchers have reported massive and increasing evidence of the presence of small plastic particles in the aquatic food chain^3, 4^. Microplastics can be directly ingested by the marine fauna^5–7^, or physically adsorb to algal species^8^ and then be ingested by zooplankton^9^, crustaceans^10^, and so on. To the best of our knowledge, specific reports on the presence of nano-sized plastic fragments in the oceans are still lacking, but this is most likely due to difficulties in their detection, since it is difficult to envisage reasons for plastic degradation to stop at micrometer sizes. The assessment of the risks associated to the interaction of micro and nanoplastics with living organisms in the marine environment is a major challenge. To date, unanswered questions concern not only the toxicity of plastic nanoparticles, but also more basic issues such as the physico-chemical characterization of micro and nanoplastics in the natural environment. Plastic degradation patterns in the ocean need to be outlined^2^, and the surface characterization of the micro and nanoplastic samples collected in the sea is largely missing.

Experiments performed in controlled laboratory conditions allow to draw a few general conclusions. First, microsized and nanosized plastic nanoparticles can accumulate in the tissues of living organisms, such as mussels, shrimps and fish^10–12^, and affect their metabolism and behavior. Second, nanoparticle size is an important factor to determine the fate of the NP in the organism, with the smaller particles having the greater potential for accumulate and translocate within the tissues of the host^11^. Third, the surface of plastic particles can adsorb and transport persistent organic pollutants, such as the hydrophobic polycyclic aromatic hydrocarbons or polychlorinated biphenlys^3, 13^, transforming the plastic particles into vehicles of toxic substances into the organism.

The study of the interactions between nanoplastics and living cells is still lacking a molecular perspective. Most every-day use plastic materials (for instance, polystyrene (PS), polyethylene (PE), polypropylene (PP)) have a strong hydrophobic character, which might drive a favorable, passive interaction of the smallest nanoparticles (below 10 nm in diameter) with lipid membranes. The favorable association between nanoplastics and lipids has been suggested to play a role in driving plastic nanoparticles to the brain^12^, via penetration of the blood-brain barrier, but no molecular study has so far explored the physical mechanisms at the basis of such an interaction.

In this paper we adopt a computational, molecular-level approach to the study of the interaction between hydrophobic polymers and model lipid bilayers. Our work builds on our previous investigation of the interaction between polystyrene nanoparticles and model lipid membranes^14^, which led us to the conclusion that the membrane physical properties – structural, dynamic, mechanical, and thermodynamic – are altered by the interaction with pristine polystyrene. Hereby we extend our study to polyethylene and polypropylene, representing the two most common plastic materials produced nowadays (30% and 19% of the European demand in 2014, respectively)^2^ and buoyant in seawater^1^. We find that PE, PP and PS can passively penetrate the hydrophobic core of model lipid membranes, and, despite their similar degree of hydrophobicity, alter membrane physical properties in different ways. In all cases, we predict very significant effects on membrane properties, both in the case of single-component, homogeneous bilayers and more complex phase-separated membranes.

## Methods

### Simulations setup

We considered the interaction of polyethylene (PE), polypropylene (PP), and polystyrene (PS) polymers with both single-component, homogeneous 1-palmitoyl-2-oleoyl-phosphatidylcholine (POPC) lipid membranes and with ternary mixtures. Single-component POPC membranes contained 2048 lipids, while ternary mixtures contained 598 dipalmitoylphosphatidylcholine (DPPC), 1598 dilinoleoyl-phosphatidilcholine (DLiPC), and 642 cholesterol (CHOL) molecules. Starting configurations for the membranes were generated with the INSANE tool^15^.

Polymer chains of the same species were placed in the water phase (not in contact with the membrane). We used different polymer concentrations, ranging from a mass ratio of 1.3% to a mass ratio of 10.9%. The length of the polymers chains ranged from a few to about one hundred monomers. A complete list of simulations performed is reported in Table 1.

**Table 1:** List of simulations performed, with lipid composition, polymer composition, and length of the production run.

System composition	Polymer/lipid mass %	Run time [μs]
POPC	—	8
POPC + 8 PP107	2.3	8
POPC + 23 PP107	6.6	8
POPC + 38 PP107	10.9	8
POPC + 8 PE80	2.3	15
POPC + 23 PE80	6.6	15
POPC + 38 PE80	10.9	15
Ternary mixture	—	10
Ternary mixture + 6 PP107	1.3	10
Ternary mixture + 44 PP107	9.7	10
Ternary mixture + 6 PE80	1.3	10
Ternary mixture + 44 PE80	9.7	8
Ternary mixture + 3 PS100	1.3	10
Ternary mixture + 19 PS100	9.7	10

The number following the name of the polymer indicates the number of units in the polymer chain represented at the coarse-grained level.

### Force fields

We used the MARTINI coarse-grained (CG) force field^16–18^ to model all the components of our systems: water, lipid membranes, and polymers. This choice allowed us to easily reach time scales of tens of microseconds, which is a requirement for the unbiased simulation of the polymer-membrane interaction. We recently developed MARTINI CG models of PS, PP, and PE that reproduce the free energy of transfer of small oligomers from water to membranes obtained in atomistic simulations^19–21^.

With the aim of validating of some of our results, we performed atomistic simulations with the OPLS-UA force field to model the polymers^22^, together with the compatible Berger lipids models^23^. All the simulations were carried out with the Gromacs^24–26^ software package (versions 4.6.7, 5.0.4, and 5.1.1).

### Simulation parameters

CG (or atomistic) simulations were performed in the NpT ensemble, with periodic boundary conditions in all dimensions. We used a leap-frog integrator with a time step of 20 fs (2 fs). The temperature was set to 310 K using the Donadio-Bussi-Parrinello thermostat^27^, with a time constant of 2 ps (0.2 ps). The pressure was set to 1 bar using Berendsen weak coupling algorithm in equilibration runs, with a time constant of 4 ps (1 ps) and the Parrinello-Rahman^28^ barostat for production runs with time constants of 8–12 ps (1 ps). Pressure control was always semi-isotropic, with z (the direction of the membrane normal) coupled independently of x and y.

### Contact analysis

To analyze phase separation in ternary systems we monitored the contacts between the different lipid species, defined as follows:

1 $${f}_{mix}=100\frac{{C}_{DLiPC-DPPC}}{{C}_{DLiPC-DPPC}+{C}_{DLiPC-DLiPC}}$$

2 $${f}_{chol}=100\frac{{C}_{CHOL-DLiPC}}{{C}_{CHOL-DLiPC}+{C}_{CHOL-DPPC}}$$

3 $${f}_{pol}=100\frac{{C}_{pol-DLiPC}}{{C}_{pol-DLiPC}+{C}_{pol-DPPC}}$$

In the above formulas, C_A–B_ is the number of contacts between molecules A and B. Lipids were considered to be in contact when the distance between their head groups (phosphate or hydroxyl) was shorter than 1.1 nm. The contacts between polymers and lipids, instead, were calculated considering all polymer and lipids beads and a threshold distance of 0.8 nm. The contact analysis was performed on the last 10 microseconds of each production run.

### Diffusion coefficient

The diffusion coefficient for the lipids, D_L_, was obtained by fitting the mean square displacement to the function f = 4 D_L_ t in the 10 ns–4 μs time window.

### Mechanical properties

The area compressibility modulus of the membrane K_A_ was calculated from the total area oscillations, using the formula:

4 $${K}_{A}={k}_{B}T\frac{{A}_{0}}{\langle {(A-{A}_{0})}^{2}\rangle }$$

where A_0_ is the average in-plane area of the membrane, A the in-plane area at each time, k_B_ the Boltzmann constant and T the temperature. The value of K_A_ was calculated with the above formula averaging over 10 microseconds.

The membrane bending modulus, K_c_, was calculated via the analysis of membrane buckling, as recently proposed by Deserno^29^ and reviewed in Bochicchio *et al*.^30^. Briefly, the POPC bilayers were buckled by applying a pressure of 6 bar in the *x* direction until a strain of 0.2 was reached (the *y* edge of the simulation box was fixed during the compression). Then, 10 microseconds simulations were carried out with constant *x* and *y* box dimensions. The value of the *xx* element of the stress tensor was then used to derive the force exerted by the membrane in the x direction, force that is in turn related to K_C_ as explained in the original reference^29^. Only the last 5 microseconds were used for averaging.

### 2D-Maps

Three kinds of 2D maps were produced: density, thickness and order parameter, using algorithms developed in Castillo^31^ *et al*. In all cases the plane of the membrane (xy) was divided into 100 × 100 bins and the quantity of interest was averaged, over time, in each grid cell. The membrane thickness is locally defined as the distance along z between the center of mass of the lipid heads in the two leaflets. The order parameter is defined as:

5 $$p=\frac{(3\langle {\cos }^{2}{\vartheta }\rangle -1)}{2}$$

where $\vartheta$ is the angle between vectors connecting two coarse-grained beads belonging to the same acyl chain and the membrane normal (Z). All the beads belonging to the acyl chains are considered, and thus the average is performed over bonds, lipids, and time.

The datasets generated during and analysed during the current study are available from the corresponding authors on reasonable request.

## Results

### Interaction between PE, PP, PS and homogeneous lipid membranes

We started our simulations from an equilibrated POPC membrane containing 2048 lipids, placing the desired quantity of polymer chains on top of the membrane, in the water phase. For all three polymers, in the initial phase of the simulations some of the polymer chains entered the membrane but most of them aggregated into a single polymer nanoparticle, diffusing in the water phase. Subsequently, the entire polymer nanoparticle entered the membrane core. Once the polymer nanoparticles make contact with the membrane, their penetration into the membrane core is fast, typically on a timescale of a few tens of ns, for all three polymers. Significantly longer time scales are required to sample an equilibrium distribution for the polymer chains in the membrane core. In the following, we analyze the equilibrated portions of the trajectories.

#### PP dissolves in the membrane, PE does not

**Figure 1:**
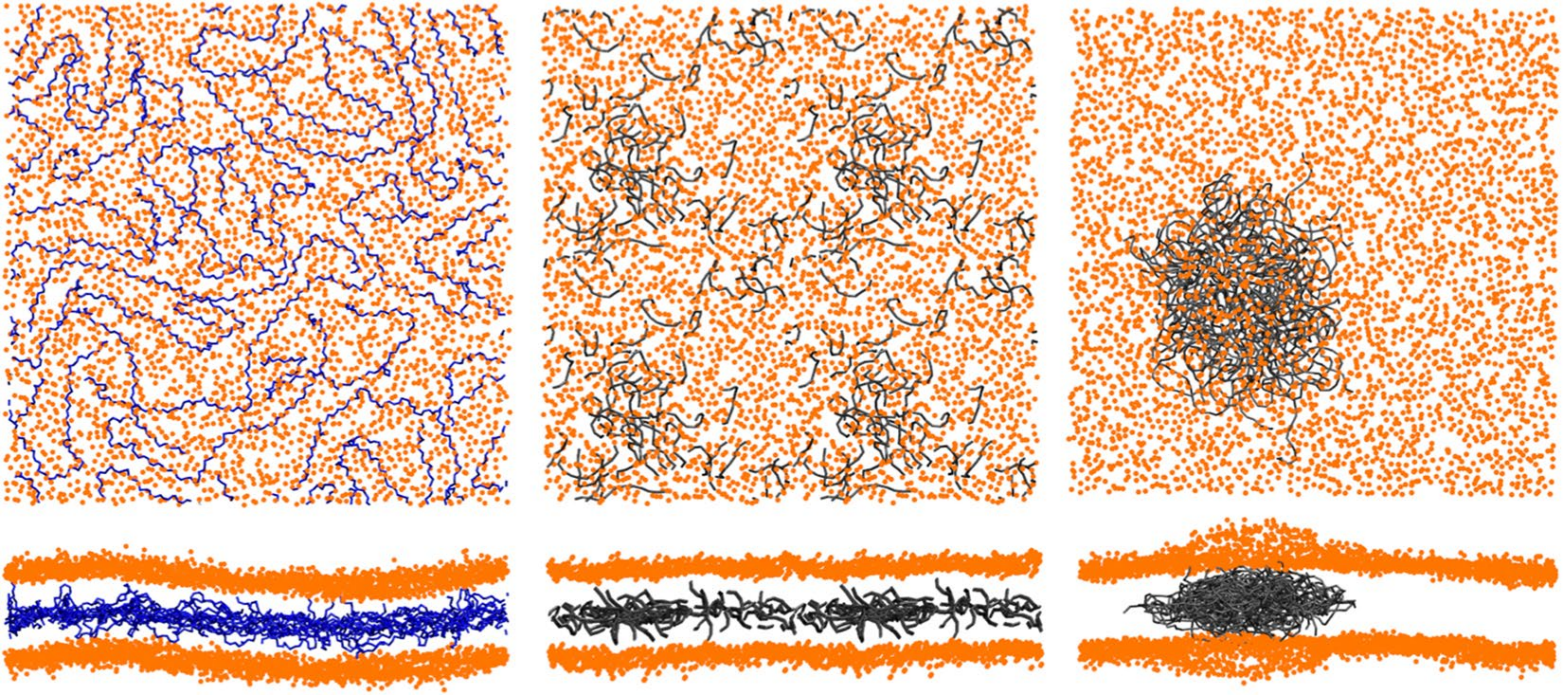
Typical distributions of the polymers inside pure POPC membranes (lipid:polymer mass ratio of 6.6%). Two views of the membrane (only head beads, in orange) are shown for each configuration: from the top and from the side. Left: PP107 chains (in blue). Middle: PE10 chains (in gray). Here the membrane contained only 512 lipids, and has been multiplied by 4 to have the same scale as other pictures. Right: PE80 chains (in gray).

PP nanoparticles dissolve upon contact with the hydrophobic membrane core, independently of the chain length and the size of the nanoparticle (the maximum size being about 7 nm in diameter). PP reaches an equilibrium distribution in which all chains are randomly dispersed in the hydrophobic core of the membrane (Fig. 1, left). Such distribution is stable on a time scale of 10 μs in CG simulations. We tested the behavior of PP in in POPC at the united atom level, as well. We prepared one entangled configuration of 2 PP107 modeled with the OPLS-UA force field and inserted it in a membrane composed of 288 POPC lipids: the chains dissolved within a time scale of a few hundred nanoseconds. We quantitatively compared the degree of dissolution predicted by the atomistic and the CG model by simulating the dissolution of an aggregate of PP13 chains in POPC, at 6.2% PP/POPC mass concentration. After dissolution, which takes place in the CG as well as in the atomist run, we calculated the number of contacts between PP monomers and found an average of 4.0+− 0.1 contacts in the CG simulation and 4.7+− 0.2 contacts in the atomistic simulation.

In CG runs (Table 1), the long PP chains (PP107) stretch out and lay flat between the two membrane leaflets. Their radius of gyration (R_g_), which is of 2.24 nm in the melt and 2.70 nm in good solvent conditions, reaches 3.40 nm in the membrane. PP chains are highly anisotropic, with a ratio between the diagonal components of their gyration tensor of 6.7:2.6:1. We had previously observed a similar behavior in the case of PS^14, 32^ (R_g_ larger than in good solvent conditions, large anisotropy).

PE behaves quite differently. For PE10 chains (the shortest chain length we simulated), a typical equilibrium distribution is shown in the middle panel of Fig. 1: here, the PE chains are dispersed inside the core of the membrane, but not in a uniform way. There are in fact always higher density zones, indicating a tendency of PE to self-aggregate. Increasing the length of the PE chains to 80 monomers, we observe that the PE chains form a single, compact cluster within the membrane core (Fig. 1, right panel). The aggregate is entangled, but the chains maintain a certain internal mobility: the aggregate is not solid. To verify that aggregation is not a kinetic effect but indeed an equilibrium state, we carried out two independent simulations starting from a configuration with 8 PE80 chains randomly dispersed within the membrane. After a few microseconds, PE80 chains were once again aggregated into a single cluster.

Since the result on PE aggregation was rather unexpected, we tested the behavior of PE in atomistic simulations, using a polymer chain length of 52 monomers and the same POPC lipid bilayer. Atomistic PE shows indeed a tendency to aggregate similar to its CG counterpart, as shown by the average number of PE-PE contacts: we find 2.6 contacts per monomer in CG simulations of PE10 (40 monomers), and 2.7 contacts per monomer in atomistic simulations of PE12 (52 monomers), which highlights a remarkable agreement between atomistic and CG models in terms of polymer aggregation in this size range.

#### PP causes moderate membrane expansion and softening

**Figure 2:**
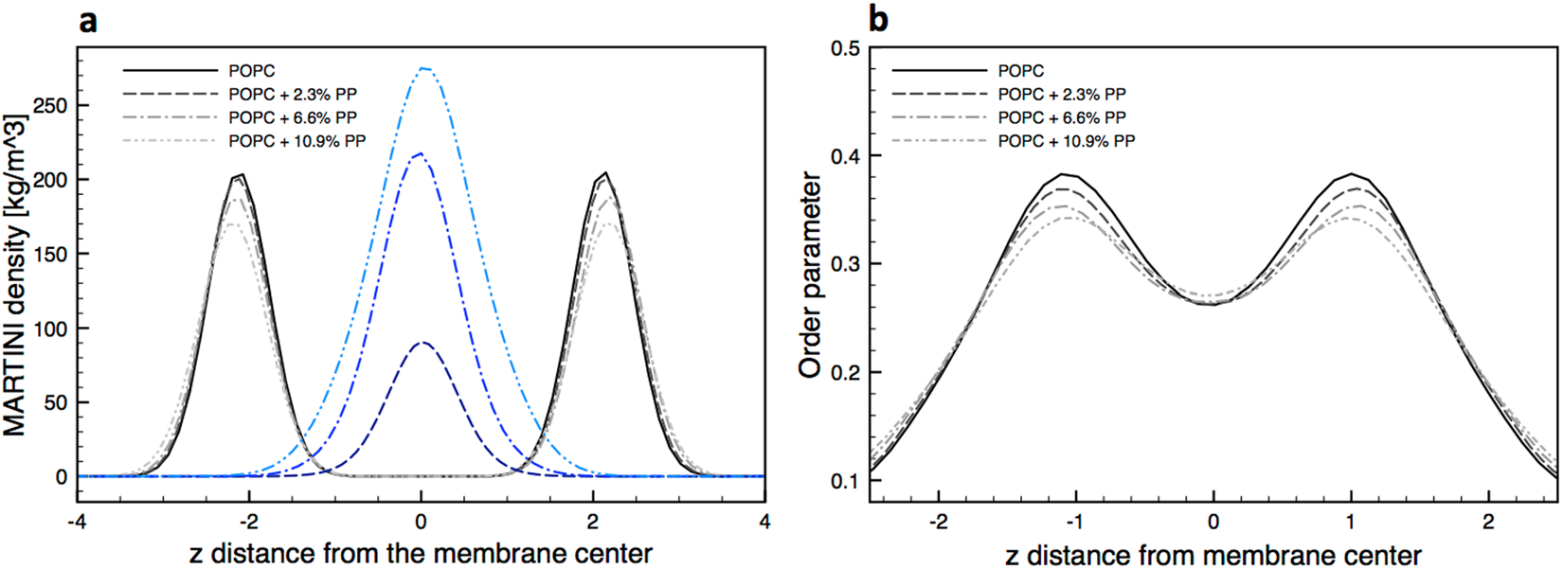
PP effect on the membrane structure. (**a**) The density profile of phosphodiester groups (black) and PP107 chains (blue) along the membrane normal. (**b**) The order parameter of lipid bonds at different PP concentrations, plotted as a function of the distance (along the membrane normal) from the membrane center.

We calculated the effect of PP on structural and dynamic properties of the POPC membrane (see Table 2 and Fig. 2). The density plot for the lipid phosphate groups (Fig. 2A) indicates little or no change in membrane thickness upon addition of the polymer. The area per lipid increases by about 3% at the highest polymer concentration. Accordingly, analysis of the order parameter of acyl chains (Fig. 2B) indicates that PP induces disorder in the membrane core, but the effect is minor. PP also reduces the diffusion coefficient of the lipids. In our previous work we had shown that PS had similar effects on the structural and dynamic properties of homogeneous POPC membranes^14^.

**Table 2:** POPC bilayer properties in the presence of different amounts of PP107.

PP/lipid mass %	D_L_ [10^−7^ cm^2^/s]	A_L_ [nm^2^]	K_A_ [mN/m]	K_c_ [k_B_T]	K_c_ [k_B_T] (PS)
0% (pure POPC)	6.20	0.650	332	36.6 +−1	
2.3%	4.77	0.656	323	—	
6.6%	3.48	0.670	312	31.5 +−1.8	19.0 +−1.8
10.9%	2.46	0.690	253	28.8 +−1.8	16.9 +−1.8

All metrics are averaged over the last 8 microseconds of simulation. Uncertainties are below 5% for D_L_, and below 1% for A_L_ and K_A_. The last column contains the bending modulus data obtained in the presence of PS, for comparison.

In terms of mechanical properties, PP makes the POPC membrane only slightly softer. We notice that the effect of PS goes in the same direction but it is significantly stronger. Two distinct methods, based on the analysis of the fluctuation spectrum (as reported in Rossi *et al*.^14^) and membrane buckling (used here), provide consistent results and indicate that the addition of 10% PS to the membrane reduces the bending modulus by a factor of two.

#### PE clusters cause long-range perturbations in membrane order

**Figure 3:**
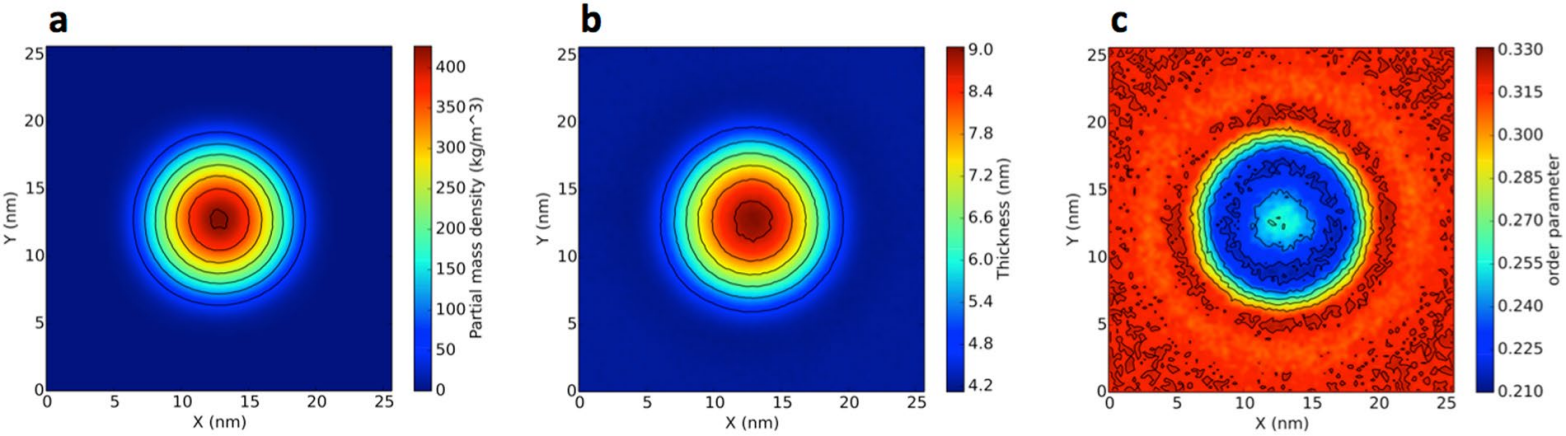
Two dimensional maps of (**a**). PE density, (**b**). Membrane thickness and (**c**). Lipid chains order parameter. The maps refer to PE80 in a pure POPC membrane. The center of mass of PE was translated to the center of the box.

For PE80, it is not possible to describe the effect on the membrane as a global modification of its properties, since PE remains aggregated and the structure of the membrane is affected only locally. For instance, the membrane thickness is strongly increased in the regions where the PE aggregates. As shown in Fig. 3, the region of increased membrane thickness matches the region with non-zero PE density. In the same region, the order parameter of lipid chains is decreased due to membrane bending and possibly higher disorder. We notice that the effect on the lipid chain order is rather long ranged, extending well beyond the radius of the polymer cluster.

### Interaction between PE, PP, PS and heterogeneous lipid membranes

**Figure 4:**
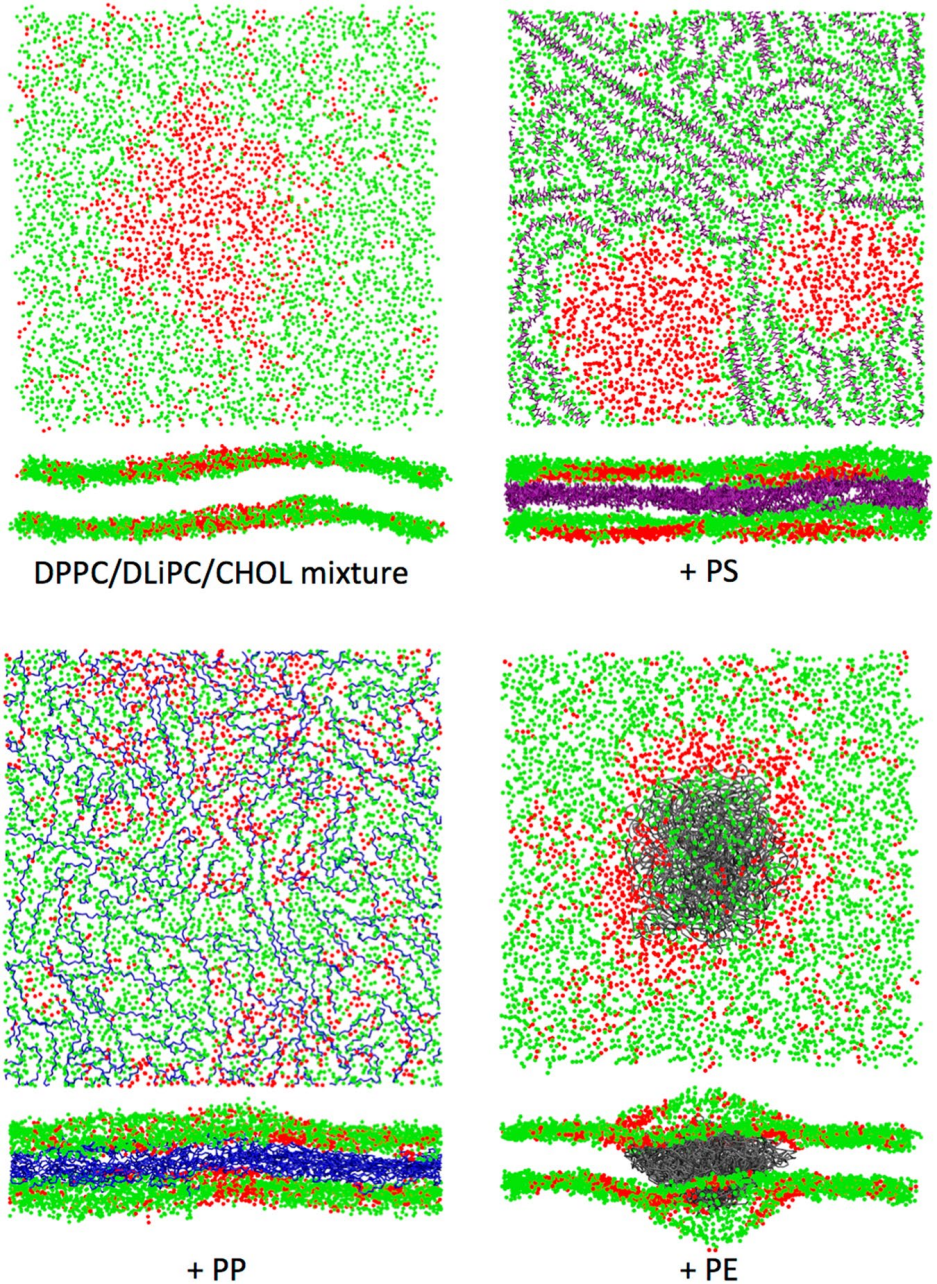
The three polymers in liquid-ordered/liquid-disordered phase separated membranes. Top left: top view of the phase separated ternary mixture. Only 1 bead per lipid is shown, from the head group. DPPC is shown in red, DLiPC in green, and CHOL is not shown for clarity. Top right: membrane + PS100 chains (in purple). Bottom left: membrane + PP107 chains (in blue). Bottom right: membrane + PE80 chains (in gray). When the polymer is present, its concentration is 9.7% (polymer/lipid mass ratio).

To set up simulations of phase-separated (Lo-Ld) membranes, we started from a mixture of DPPC, DLiPC and cholesterol (Chol) with 20% DPPC, 59% DLiPC, and 21% Chol. We allowed the system to freely evolve for 10 microseconds, after which the phase separation into Lo and Ld was completed. We monitored the evolution and the stability of the phase separation by calculating the percentage of contacts between the different lipid species as a function of time (see the Methods section). A typical snapshot of the equilibrium configuration is shown in Fig. 4: a liquid ordered island (mainly composed by DPPC and Chol) is surrounded by the liquid disordered phase (mainly composed by DLiPC).

After the stabilization of the contact metrics, polymer chains (PE, PP, and PS) were introduced into the membrane core in a randomly dispersed configuration. The systems were equilibrated and then allowed to evolve in pre-production runs until no drift was detected in the contact metrics. The whole process required 5 to 20 microseconds, depending on the nature and the amount of polymer.

The effect of PS on the phase separation reported in our previous work^14^ was based on simulations featuring stripe-shaped, periodic Lo-Ld phases. Here we carried out simulations using the same set-up for PS, PP, and PE, with larger Ld domains and no periodic stripes (see Fig. 4). The results confirm our previous conclusions^14, 33^: PS favors phase separation, decreasing the number of contacts between unsaturated and saturated lipids. PS chains partition into the Ld phase (Fig. 4, top right panel), further depleting the Ld phase of cholesterol molecules. These effects are quantified by the analysis reported in Table 3. At both polymer concentrations, the fraction of DPPC-DLiPC contacts (f_mix_) and the fraction of Chol-DLiPC contacts (f_chol_) decrease. At the same time, the fraction of polymer-DLiPC contacts (f_pol_) is much higher than expected for a random distribution (73%), indicating a strong preference of PS for partitioning into the Ld phase. These results are consistent with our previous simulations on aromatic compounds of different size, all showing a strong preference for the Ld phase and all stabilizing phase separation in similar ternary lipid mixtures^33^.

**Table 3:** Influence of the polymers chains on phase separation in ternary systems DPPC/DLiPC/Chol.

Polymer/lipid mass%	f_mix_	f_chol_	f_pol_
0%	12.4	48.2	
PS 1.5%	10.2	44.0	98.7
PS 9.7%	6.5	32.0	98.1
PP 1.3%	14.5	52.5	50.4
PP 9.7%	19.6	58.6	68.2
PE1.3%	12.4	47.7	56.6
PE 9.7%	16.0	50.0	48.7

f_mix_, f_chol_ and f_pol_ indicate the fraction of DPPC-DLiPC contacts, Chol-DLiPC contacts, and polymer-DLiPC contacts respectively. In the case of ideal mixing, the expected values for f_mix_, f_chol_ and f_pol_ are 0.27, 0.73, and 0.73 (the lipid mixture has a DPPC:DLiPC ratio of 27:73). Statistical uncertainties are below 5%, except for f_pol_ in the case of PE (10% uncertainty).

As for PP, a completely different behavior is observed. PP chains dissolve both in the Lo and in the Ld phase, with a slight preference for DPPC. Moreover, PP perturbs phase separation and increases lipid mixing (as shown by higher values of f_mix_ and f_chol_ compared to system without polymers), as shown in Fig. 4.

**Figure 5:**
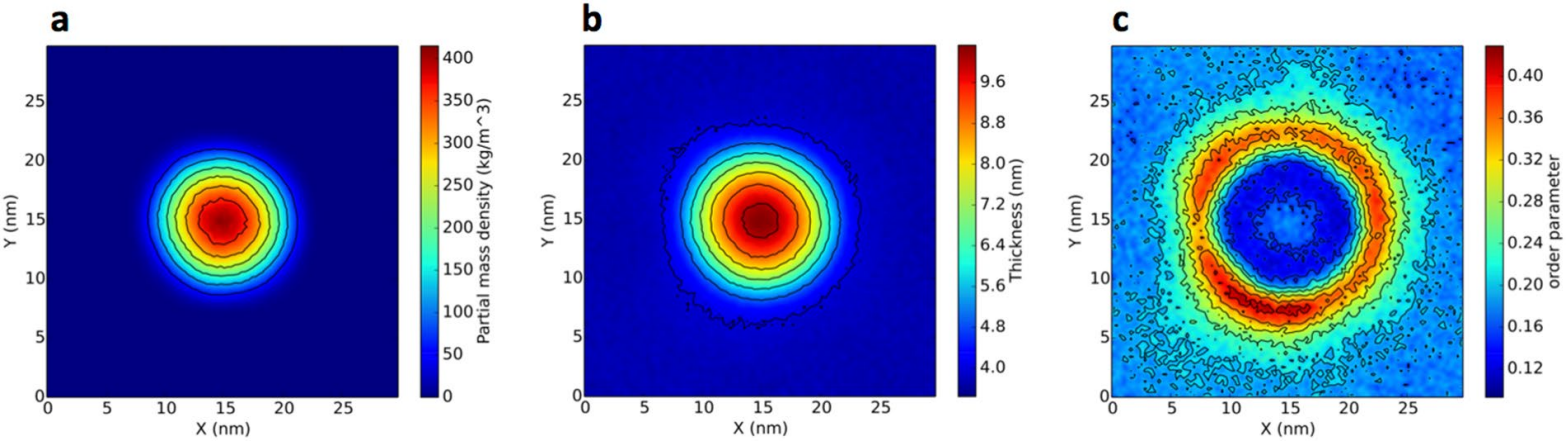
PE in the ternary mixture of DPPC, DIPC and cholesterol. Bi-dimensional maps of the (**a**). PE density, (**b**). Membrane thickness and (**c**). Lipid tails order parameter.

**Figure 6:**
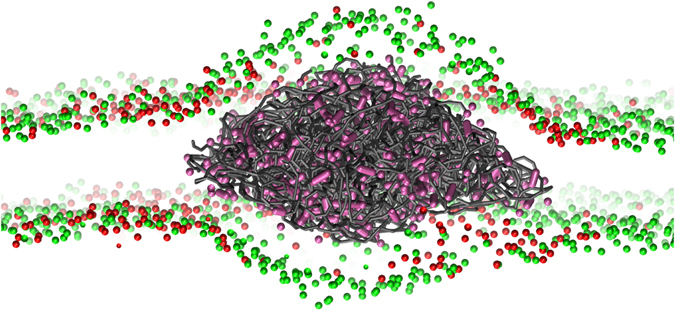
Snapshot from a simulation of the ternary system + PE80 (9.7% in mass), highlighting the presence of cholesterol within the PE aggregate. For phospholipids, only the head groups are shown: DPPC in red, DLiPC in green. The PE chains are in gray, and Chol beads within 0.5 nm of PE are shown in pink.

Let us now analyze the case of PE. In phase-separated lipid bilayers, PE shows a clear tendency to aggregate, as already observed in pure POPC bilayers. However, the equilibrium configuration of the system shows some interesting features. First of all, at the lower concentration (1.3% in mass), the polymer has no effect on phase separation, while at the higher concentration (9.7%) it promotes mixing, although more weakly than PP – an Lo and an Ld phase are recognizable at both polymer concentrations. Second, as shown in Fig. 4, the PE cluster is covered mainly by DLiPC (Ld phase), and the DLiPC-rich phase is surrounded mostly by DPPC lipids, forming roughly an annulus (ring) around the DLiPC circle. This lipid distribution is stable for 20 microseconds; the DLiPC central circle and DPPC ring are clearly visible both at low and high polymer concentration. The DPPC ring spans about 3-nm in radius, and the bilayer in the annulus region is thicker and more ordered than the surrounding DLiPC-rich membrane (Fig. 5). Third, some cholesterol molecules can be found inside the PE aggregate (Fig. 6). Cholesterol is not kinetically trapped in the PE cluster, in fact it diffuses in and out the PE cluster during the simulation, remaining within the PE cluster only for a few nanoseconds. The number of cholesterol-PE contacts fluctuates without any drift over time scales of tens of microseconds. The presence of cholesterol inside the PE cluster leads to cholesterol depletion from the Lo phase, that is probably the reason for the decreased stability of the Lo phase itself and the increase in lipid mixing caused by the presence of PE.

## Conclusions

The interaction between polymer plastics and biological membranes is relevant for environmental and health sciences, but very little is understood at the molecular level. In the present work we used molecular simulations at the coarse-grained level to examine the interaction between model lipid membranes and three very common polymers, namely polyethylene, polypropylene, and polystyrene, which are the most abundant plastic polymers found in ocean debris. Due to their hydrophobic character and to the small size of the nanoparticles, in all three cases polymer particles enter easily lipid membranes, and affect their properties. Remarkable differences are observed in the behavior of the three polymers once inside lipid membranes: PE shows a strong tendency to self-aggregate within lipid bilayers, forming lens-shaped clusters, while PS and PP dissolve completely. To our knowledge, no experimental study has been devoted so far to the localization of PE, PP or PS chains in plasma or model lipid membranes. As for PS, our results are qualitatively coherent with the freeze-fracture microscopy observations by Radlinska^32^ *et al*., which indicate that PS-sulfonate chains, at the lowest degree of sulfonation (30%), indeed swell in the midplane of synthetic nonionic surfactant bilayers. As for PE, oil lenses have been experimentally observed^34^, but for short alkanes and in 1,2-diphytanoyl-sn-glycero-3-phosphocholine bilayers. We calculated changes in structural and mechanical properties of the membrane only in the case of PS and PP, since the membrane in the presence of PE is no longer homogeneous. In the case of single-component, homogeneous POPC membranes, we observed very significant changes in membrane elastic properties, with a reduction in the bending moduli by a factor of two for PS and by about 20% for PP (at 10.9% polymer concentration). Lipid diffusion is also reduced in the presence of the polymers. In the case of phase-separated ternary lipid mixtures, three distinct types of behavior are observed: PS localizes to the Ld phase and stabilizes phase separation, PP has a weak preference for the Lo phase and destabilizes phase separation, while PE yields a peculiar lipid distribution, with the polymer cluster coated by the Ld phase and the Ld phase surrounded by an annulus of Lo phase. PE reduces the cholesterol concentration in the Lo phase, which contributes to the destabilization of phase separation. In all cases, the effect of the polymer on phase separation is significant, and calls for experimental investigations in model and in biological systems.
